# *N*-[2-(Amino­carbon­yl)phen­yl]-4-hydr­oxy-2-methyl-2*H*-1,2-benzothia­zine-3-carboxamide 1,1-dioxide

**DOI:** 10.1107/S1600536809038951

**Published:** 2009-09-30

**Authors:** Muhammad Nadeem Arshad, Muhammad Zia-ur-Rehman, Islam Ullah Khan

**Affiliations:** a Department of Chemistry, Government College University, Lahore 54000, Pakistan; b Applied Chemistry Research Centre, PCSIR Laboratories Complex, Ferozpure Road, Lahore 54600, Pakistan

## Abstract

In the title compound, C_17_H_15_N_3_O_5_S, the thia­zine ring adopts a distorted half-chair conformation. The mol­ecular structure is stabilized by intra­molecular N—H⋯O, N—H⋯N and O—H⋯O hydrogen bonding. Pairs of mol­ecules are bound together as centrosymmetric dimers through N—H⋯O hydrogen bonds.

## Related literature

For the synthesis of related mol­ecules, see: Braun (1923 ▶); Ahmad *et al.* (2008 ▶); Zia-ur-Rehman *et al.* (2005 ▶, 2009 ▶). For the biological activity of 1,2-benzothia­zine 1,1-dioxides, see: Bihovsky *et al.* (2004 ▶); Turck *et al.* (1996 ▶); Zia-ur-Rehman *et al.* (2006 ▶). For similar mol­ecules, see: Kojić-Prodić & Rużić-Toroš (1982 ▶); Siddiqui *et al.* (2009 ▶); Weast *et al.* (1984 ▶); Zia-ur-Rehman *et al.* (2007 ▶).
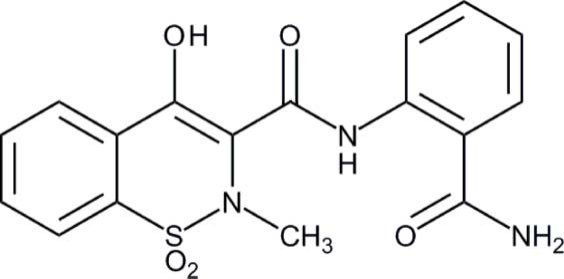


## Experimental

### 

#### Crystal data

C_17_H_15_N_3_O_5_S
*M* *_r_* = 373.38
Monoclinic, 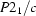

*a* = 8.1377 (6) Å
*b* = 7.0515 (6) Å
*c* = 29.069 (2) Å
β = 96.502 (3)°
*V* = 1657.3 (2) Å^3^
*Z* = 4
Mo *K*α radiation
μ = 0.23 mm^−1^
*T* = 296 K
0.39 × 0.25 × 0.11 mm

#### Data collection

Bruker APEXII CCD area-detector diffractometer
Absorption correction: multi-scan (*SADABS*; Sheldrick, 2007 ▶) *T*_min_ = 0.915, *T*_max_ = 0.975
16109 measured reflections
3753 independent reflections
3011 reflections with *I* > 2σ(*I*)
*R*_int_ = 0.039

#### Refinement

*R*[*F* ^2^ > 2σ(*F* ^2^)] = 0.087
*wR*(*F* ^2^) = 0.213
*S* = 1.09
3753 reflections
237 parameters
H-atom parameters constrained
Δρ_max_ = 0.38 e Å^−3^
Δρ_min_ = −0.40 e Å^−3^

### 

Data collection: *APEX2* (Bruker, 2007 ▶); cell refinement: *SAINT* (Bruker, 2007 ▶); data reduction: *SAINT*; program(s) used to solve structure: *SHELXS97* (Sheldrick, 2008 ▶); program(s) used to refine structure: *SHELXL97* (Sheldrick, 2008 ▶); molecular graphics: *PLATON* (Spek, 2009 ▶) and *Mercury* (Macrae *et al.*, 2006 ▶); software used to prepare material for publication: *WinGX* (Farrugia, 1999 ▶) and *PLATON*.

## Supplementary Material

Crystal structure: contains datablocks I, global. DOI: 10.1107/S1600536809038951/bt5073sup1.cif

Structure factors: contains datablocks I. DOI: 10.1107/S1600536809038951/bt5073Isup2.hkl

Additional supplementary materials:  crystallographic information; 3D view; checkCIF report

## Figures and Tables

**Table 1:** Hydrogen-bond geometry (Å, °)

*D*—H⋯*A*	*D*—H	H⋯*A*	*D*⋯*A*	*D*—H⋯*A*
O1—H1⋯O4	0.82	1.85	2.569 (5)	145
N2—H2⋯O5	0.86	1.92	2.607 (5)	136
N2—H2⋯N1	0.86	2.28	2.728 (5)	113
N3—H3*A*⋯O5^i^	0.86	2.22	2.941 (6)	141

Symmetry code: (i) 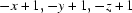.

**Fig. 1.**
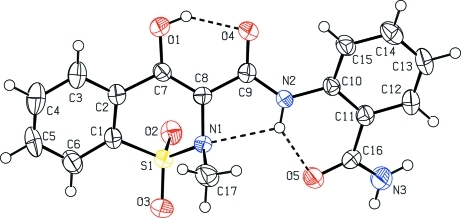
The molecular structure of the title compound with displacement ellipsoids at the 50% probability level.

**Fig. 2.**
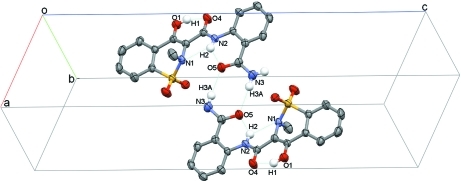
Perspective view of the three-dimensional crystal packing showing hydrogen-bonded interactions (dashed lines). H atoms not involved in hydrogen bonding have been omitted for clarity.

## supplementary crystallographic information

### Comment

Owing to the verstaile applications of 1,2-benzothiazine 1,1-dioxides, considerable attention has been given to their synthesis since their very first synthesis (Braun, 1923). Among these, Piroxicam (Zia-ur-Rehman *et al.*, 2005), and Meloxicam (Turck *et al.*, 1996) are familiar for their analgesic action and are being used world wide as non-steroidal anti-inflammatory drugs (NSAIDs).  Some of the 3,4-dihydro-1,2-benzothiazine-3-carboxylate 1,1-dioxide α-ketomide and P(2)—P(3) peptide mimetic aldehyde compounds act as potent calpain I inhibitors (Bihovsky *et al.*, 2004) while 1,2-benzothiazin-3-yl-quinazolin-4(3*H*)-ones possess anti-bacterial properties (Zia-ur-Rehman *et al.*, 2006).

In continuation of our work on the synthesis (Zia-ur-Rehman *et al.*, 2006, biological activity (Zia-ur-Rehman *et al.*, 2009) and crystal structures (Zia-ur-Rehman *et al.*, 2007; Ahmad *et al.*, 2008, Siddiqui *et al.*, 2009) of various 1,2-benzothiazine-1,1-dioxides, we herein report the crystal structure of the title compound (I) (Scheme and figure 1). Like its already reported dimethylsulfoxide solvate analogue (Zia-ur-Rehman *et al.*, 2007), thiazine ring involving two double bonds, exhibits sofa conformation; with S1/C1/C2/C7 relatively planar and N1 showing significant departure from plane due to its pyramidal geometry. The enolic hydrogen on O1 is involved in intramolecular hydrogen bonding [O1—H1···O4] with the carbonyl oxygen at C9 giving rise to a six-membered hydrogen bond ring (Table 1). Atom H2 forms hydrogen bonds with both N1 and O5 giving rise to five and six-membered hydrogen bond rings respectively. The C1—S1 [1.755 Å] bond is shorter than a normal C—S single bond (1.81–2.55 Å) (Weast *et al.*, 1984) due to partial double bond character and is in agreement with similar molecules (Kojić-Prodić & Rużić-Toroš, 1982). Each molecule is centrosymmetrically linked to its adjacent one forming a dimer through intermolecular [N—H3B···O5] hydrogen bonds (Fig. 2).

### Experimental

*N*-[2-(Aminocarbonyl)phenyl]-4-hydroxy-2-methyl-2*H*-1,2-benzothiazine- 3-carboxamide 1,1-dioxide was synthesized according to a literature method (Zia-ur-Rehman *et al.*, 2006). Suitable crystals were obtained by dissoving the compound in chloroform followed by slow evaporation at room temperature.The compound was dissolved in a mixture of methanol and DMSO (80:20 *v*/*v*) at room temperature. Crystals were obtained by slow evaporation and dried under high vacuum.

### Refinement

All hydrogen atoms were identified in the difference map. They were refined using a riding model with O—H =  0.84 Å, N—H =  0.86 Å, C_methyl_—H 0.98 Å and C_aromatic_—H = 0.95 Å. and  U(H) set to 1.2Ueq of the parent atoms  or set to 1.5Ueq(C_methyl_).

### Crystal data

**Table:**

C_17_H_15_N_3_O_5_S	*F*(000) = 776
*M**_r_* = 373.38	*D*_x_ = 1.496 Mg m^−^^3^
Monoclinic, *P*2_1_/*c*	Mo *K*α radiation, λ = 0.71073 Å
Hall symbol: -P 2ybc	Cell parameters from 6164 reflections
*a* = 8.1377 (6) Å	θ = 2.5–27.1°
*b* = 7.0515 (6) Å	µ = 0.23 mm^−^^1^
*c* = 29.069 (2) Å	*T* = 296 K
β = 96.502 (3)°	Needles, colourless
*V* = 1657.3 (2) Å^3^	0.39 × 0.25 × 0.11 mm
*Z* = 4	

### Data collection

**Table:**

Bruker APEXII CCD area-detector diffractometer	3753 independent reflections
Radiation source: fine-focus sealed tube	3011 reflections with *I* > 2σ(*I*)
graphite	*R*_int_ = 0.039
φ and ω scans	θ_max_ = 27.5°, θ_min_ = 2.5°
Absorption correction: multi-scan (*SADABS*; Sheldrick, 2007)	*h* = −10→10
*T*_min_ = 0.915, *T*_max_ = 0.975	*k* = −9→9
16109 measured reflections	*l* = −32→37

### Refinement

**Table:**

Refinement on *F*^2^	Primary atom site location: structure-invariant direct methods
Least-squares matrix: full	Secondary atom site location: difference Fourier map
*R*[*F*^2^ > 2σ(*F*^2^)] = 0.087	Hydrogen site location: inferred from neighbouring sites
*wR*(*F*^2^) = 0.213	H-atom parameters constrained
*S* = 1.09	*w* = 1/[σ^2^(*F*_o_^2^) + (0.0335*P*)^2^ + 9.427*P*] where *P* = (*F*_o_^2^ + 2*F*_c_^2^)/3
3753 reflections	(Δ/σ)_max_ < 0.001
237 parameters	Δρ_max_ = 0.38 e Å^−^^3^
0 restraints	Δρ_min_ = −0.40 e Å^−^^3^

### Special details

**Table:**

Geometry. All e.s.d.'s (except the e.s.d. in the dihedral angle between two l.s. planes) are estimated using the full covariance matrix. The cell e.s.d.'s are taken into account individually in the estimation of e.s.d.'s in distances, angles and torsion angles; correlations between e.s.d.'s in cell parameters are only used when they are defined by crystal symmetry. An approximate (isotropic) treatment of cell e.s.d.'s is used for estimating e.s.d.'s involving l.s. planes.
Refinement. Refinement of *F*^2^ against ALL reflections. The weighted *R*-factor *wR* and goodness of fit *S* are based on *F*^2^, conventional *R*-factors *R* are based on *F*, with *F* set to zero for negative *F*^2^. The threshold expression of *F*^2^ > σ(*F*^2^) is used only for calculating *R*-factors(gt) *etc*. and is not relevant to the choice of reflections for refinement. *R*-factors based on *F*^2^ are statistically about twice as large as those based on *F*, and *R*- factors based on ALL data will be even larger.

### Fractional atomic coordinates and isotropic or equivalent isotropic displacement parameters (Å^2^)

**Table:**

	*x*	*y*	*z*	*U*_iso_*/*U*_eq_	
S1	0.35684 (14)	0.5031 (2)	0.33289 (4)	0.0343 (3)	
O2	0.2977 (5)	0.6850 (5)	0.34450 (12)	0.0427 (9)	
O3	0.5306 (4)	0.4668 (7)	0.33922 (13)	0.0538 (11)	
O4	−0.1641 (4)	0.3152 (6)	0.38428 (12)	0.0480 (10)	
O5	0.3670 (4)	0.3198 (6)	0.47838 (12)	0.0484 (10)	
N1	0.2640 (5)	0.3427 (6)	0.36214 (13)	0.0310 (9)	
N2	0.0743 (5)	0.2848 (6)	0.43247 (12)	0.0306 (8)	
H2	0.1801	0.2826	0.4328	0.037*	
N3	0.3804 (6)	0.4241 (8)	0.55160 (15)	0.0534 (13)	
H3A	0.4817	0.4585	0.5513	0.064*	
H3B	0.3319	0.4407	0.5761	0.064*	
O1	−0.1513 (4)	0.3895 (6)	0.29823 (12)	0.0436 (9)	
H1	−0.1968	0.3747	0.3218	0.065*	
C1	0.2765 (6)	0.4493 (7)	0.27578 (16)	0.0329 (10)	
C2	0.1069 (6)	0.4046 (7)	0.26901 (16)	0.0322 (10)	
C3	0.0347 (8)	0.3766 (8)	0.22406 (17)	0.0445 (13)	
H3	−0.0780	0.3516	0.2183	0.053*	
C4	0.1301 (9)	0.3861 (9)	0.18781 (19)	0.0577 (17)	
H4	0.0805	0.3685	0.1577	0.069*	
C5	0.2966 (9)	0.4210 (10)	0.19540 (19)	0.0580 (17)	
H5	0.3594	0.4221	0.1706	0.070*	
C6	0.3718 (7)	0.4546 (9)	0.23979 (19)	0.0470 (14)	
H6	0.4845	0.4802	0.2451	0.056*	
C7	0.0124 (6)	0.3801 (7)	0.30885 (16)	0.0314 (10)	
C8	0.0874 (6)	0.3479 (7)	0.35254 (16)	0.0308 (10)	
C9	−0.0106 (6)	0.3133 (7)	0.39093 (15)	0.0315 (10)	
C10	0.0150 (6)	0.2579 (6)	0.47583 (15)	0.0287 (9)	
C11	0.1236 (6)	0.2888 (7)	0.51660 (16)	0.0323 (10)	
C12	0.0636 (7)	0.2643 (7)	0.55876 (17)	0.0402 (12)	
H12	0.1350	0.2810	0.5858	0.048*	
C13	−0.0980 (7)	0.2160 (8)	0.56205 (18)	0.0441 (13)	
H13	−0.1358	0.2046	0.5909	0.053*	
C14	−0.2039 (7)	0.1845 (8)	0.5223 (2)	0.0448 (13)	
H14	−0.3134	0.1514	0.5245	0.054*	
C15	−0.1485 (6)	0.2019 (7)	0.47945 (17)	0.0374 (11)	
H15	−0.2197	0.1764	0.4528	0.045*	
C16	0.2986 (6)	0.3449 (8)	0.51412 (16)	0.0368 (11)	
C17	0.3393 (7)	0.1508 (9)	0.3632 (2)	0.0521 (15)	
H17A	0.3025	0.0790	0.3882	0.078*	
H17B	0.4576	0.1619	0.3678	0.078*	
H17C	0.3065	0.0873	0.3344	0.078*	

### Atomic displacement parameters (Å^2^)

**Table:**

	*U*^11^	*U*^22^	*U*^33^	*U*^12^	*U*^13^	*U*^23^
S1	0.0273 (5)	0.0450 (7)	0.0309 (6)	−0.0003 (5)	0.0047 (4)	0.0006 (5)
O2	0.048 (2)	0.041 (2)	0.0391 (19)	−0.0070 (17)	0.0065 (16)	−0.0037 (16)
O3	0.0284 (18)	0.084 (3)	0.049 (2)	0.000 (2)	0.0067 (16)	0.010 (2)
O4	0.0289 (18)	0.079 (3)	0.0368 (19)	0.0027 (19)	0.0076 (14)	0.0050 (19)
O5	0.0350 (19)	0.075 (3)	0.0354 (19)	−0.0012 (19)	0.0067 (15)	−0.0072 (19)
N1	0.0278 (19)	0.038 (2)	0.0276 (19)	0.0065 (17)	0.0039 (15)	0.0006 (16)
N2	0.0260 (19)	0.039 (2)	0.0280 (19)	0.0001 (17)	0.0063 (15)	0.0053 (17)
N3	0.042 (3)	0.082 (4)	0.036 (2)	−0.006 (3)	0.0011 (19)	−0.009 (2)
O1	0.0307 (18)	0.065 (3)	0.0335 (18)	−0.0013 (18)	−0.0011 (14)	0.0024 (18)
C1	0.040 (3)	0.032 (2)	0.028 (2)	0.001 (2)	0.0079 (19)	0.0016 (18)
C2	0.037 (2)	0.031 (2)	0.029 (2)	−0.001 (2)	0.0064 (19)	0.0005 (19)
C3	0.063 (4)	0.042 (3)	0.029 (2)	−0.005 (3)	0.005 (2)	−0.002 (2)
C4	0.087 (5)	0.056 (4)	0.029 (3)	−0.013 (3)	0.004 (3)	−0.005 (3)
C5	0.076 (4)	0.071 (4)	0.031 (3)	−0.005 (4)	0.024 (3)	−0.004 (3)
C6	0.046 (3)	0.057 (4)	0.041 (3)	−0.003 (3)	0.016 (2)	−0.001 (3)
C7	0.030 (2)	0.034 (2)	0.030 (2)	0.0008 (19)	0.0067 (18)	−0.0015 (19)
C8	0.028 (2)	0.034 (2)	0.030 (2)	0.0032 (19)	0.0029 (17)	0.0000 (19)
C9	0.034 (2)	0.032 (2)	0.029 (2)	0.0000 (19)	0.0034 (18)	−0.0003 (19)
C10	0.032 (2)	0.026 (2)	0.028 (2)	0.0039 (18)	0.0082 (18)	0.0025 (18)
C11	0.039 (3)	0.028 (2)	0.031 (2)	0.004 (2)	0.0072 (19)	0.0029 (19)
C12	0.058 (3)	0.035 (3)	0.029 (2)	0.002 (2)	0.011 (2)	0.001 (2)
C13	0.063 (4)	0.039 (3)	0.035 (3)	0.000 (3)	0.023 (2)	0.003 (2)
C14	0.045 (3)	0.039 (3)	0.054 (3)	−0.002 (2)	0.021 (3)	0.005 (3)
C15	0.036 (3)	0.039 (3)	0.039 (3)	0.000 (2)	0.012 (2)	0.007 (2)
C16	0.034 (3)	0.044 (3)	0.031 (2)	0.006 (2)	−0.0011 (19)	0.003 (2)
C17	0.052 (3)	0.049 (3)	0.058 (4)	0.020 (3)	0.020 (3)	0.014 (3)

### Geometric parameters (Å, °)

**Table:**

S1—O2	1.424 (4)	C4—C5	1.371 (9)
S1—O3	1.428 (4)	C4—H4	0.9300
S1—N1	1.648 (4)	C5—C6	1.384 (8)
S1—C1	1.755 (5)	C5—H5	0.9300
O4—C9	1.242 (6)	C6—H6	0.9300
O5—C16	1.246 (6)	C7—C8	1.364 (6)
N1—C8	1.433 (6)	C8—C9	1.464 (6)
N1—C17	1.484 (7)	C10—C15	1.403 (6)
N2—C9	1.337 (6)	C10—C11	1.413 (6)
N2—C10	1.412 (5)	C11—C12	1.380 (6)
N2—H2	0.8600	C11—C16	1.488 (7)
N3—C16	1.333 (6)	C12—C13	1.372 (8)
N3—H3A	0.8600	C12—H12	0.9300
N3—H3B	0.8600	C13—C14	1.378 (8)
O1—C7	1.335 (5)	C13—H13	0.9300
O1—H1	0.8200	C14—C15	1.379 (7)
C1—C6	1.372 (7)	C14—H14	0.9300
C1—C2	1.407 (7)	C15—H15	0.9300
C2—C3	1.385 (7)	C17—H17A	0.9600
C2—C7	1.471 (6)	C17—H17B	0.9600
C3—C4	1.379 (8)	C17—H17C	0.9600
C3—H3	0.9300		
			
O2—S1—O3	119.2 (3)	O1—C7—C2	114.2 (4)
O2—S1—N1	108.0 (2)	C8—C7—C2	122.3 (4)
O3—S1—N1	108.4 (2)	C7—C8—N1	121.2 (4)
O2—S1—C1	108.6 (2)	C7—C8—C9	120.8 (4)
O3—S1—C1	109.9 (2)	N1—C8—C9	117.9 (4)
N1—S1—C1	101.3 (2)	O4—C9—N2	123.4 (4)
C8—N1—C17	115.4 (4)	O4—C9—C8	120.3 (4)
C8—N1—S1	113.0 (3)	N2—C9—C8	116.3 (4)
C17—N1—S1	115.1 (3)	C15—C10—N2	121.8 (4)
C9—N2—C10	129.2 (4)	C15—C10—C11	119.3 (4)
C9—N2—H2	115.4	N2—C10—C11	118.9 (4)
C10—N2—H2	115.4	C12—C11—C10	118.4 (5)
C16—N3—H3A	120.0	C12—C11—C16	120.9 (5)
C16—N3—H3B	120.0	C10—C11—C16	120.8 (4)
H3A—N3—H3B	120.0	C13—C12—C11	122.1 (5)
C7—O1—H1	109.5	C13—C12—H12	119.0
C6—C1—C2	122.0 (5)	C11—C12—H12	119.0
C6—C1—S1	122.2 (4)	C12—C13—C14	119.7 (5)
C2—C1—S1	115.8 (3)	C12—C13—H13	120.2
C3—C2—C1	118.0 (5)	C14—C13—H13	120.2
C3—C2—C7	121.5 (5)	C13—C14—C15	120.4 (5)
C1—C2—C7	120.5 (4)	C13—C14—H14	119.8
C4—C3—C2	119.9 (6)	C15—C14—H14	119.8
C4—C3—H3	120.1	C14—C15—C10	120.2 (5)
C2—C3—H3	120.1	C14—C15—H15	119.9
C5—C4—C3	121.2 (5)	C10—C15—H15	119.9
C5—C4—H4	119.4	O5—C16—N3	120.8 (5)
C3—C4—H4	119.4	O5—C16—C11	121.6 (4)
C4—C5—C6	120.4 (5)	N3—C16—C11	117.6 (4)
C4—C5—H5	119.8	N1—C17—H17A	109.5
C6—C5—H5	119.8	N1—C17—H17B	109.5
C1—C6—C5	118.5 (5)	H17A—C17—H17B	109.5
C1—C6—H6	120.7	N1—C17—H17C	109.5
C5—C6—H6	120.7	H17A—C17—H17C	109.5
O1—C7—C8	123.6 (4)	H17B—C17—H17C	109.5
			
O2—S1—N1—C8	58.6 (4)	O1—C7—C8—C9	2.6 (8)
O3—S1—N1—C8	−171.0 (3)	C2—C7—C8—C9	−176.4 (4)
C1—S1—N1—C8	−55.4 (4)	C17—N1—C8—C7	−96.6 (6)
O2—S1—N1—C17	−165.8 (4)	S1—N1—C8—C7	38.8 (6)
O3—S1—N1—C17	−35.4 (4)	C17—N1—C8—C9	82.0 (5)
C1—S1—N1—C17	80.2 (4)	S1—N1—C8—C9	−142.6 (4)
O2—S1—C1—C6	106.1 (5)	C10—N2—C9—O4	−2.0 (8)
O3—S1—C1—C6	−25.9 (5)	C10—N2—C9—C8	176.5 (4)
N1—S1—C1—C6	−140.4 (5)	C7—C8—C9—O4	−0.9 (8)
O2—S1—C1—C2	−72.5 (4)	N1—C8—C9—O4	−179.6 (5)
O3—S1—C1—C2	155.5 (4)	C7—C8—C9—N2	−179.5 (5)
N1—S1—C1—C2	41.0 (4)	N1—C8—C9—N2	1.9 (7)
C6—C1—C2—C3	−3.9 (8)	C9—N2—C10—C15	19.3 (8)
S1—C1—C2—C3	174.7 (4)	C9—N2—C10—C11	−160.5 (5)
C6—C1—C2—C7	173.4 (5)	C15—C10—C11—C12	−0.6 (7)
S1—C1—C2—C7	−8.0 (6)	N2—C10—C11—C12	179.2 (4)
C1—C2—C3—C4	2.4 (8)	C15—C10—C11—C16	178.9 (4)
C7—C2—C3—C4	−174.9 (5)	N2—C10—C11—C16	−1.3 (7)
C2—C3—C4—C5	0.7 (10)	C10—C11—C12—C13	−1.7 (8)
C3—C4—C5—C6	−2.4 (11)	C16—C11—C12—C13	178.8 (5)
C2—C1—C6—C5	2.2 (9)	C11—C12—C13—C14	2.2 (8)
S1—C1—C6—C5	−176.3 (5)	C12—C13—C14—C15	−0.2 (8)
C4—C5—C6—C1	0.9 (10)	C13—C14—C15—C10	−2.1 (8)
C3—C2—C7—O1	−20.1 (7)	N2—C10—C15—C14	−177.3 (5)
C1—C2—C7—O1	162.7 (5)	C11—C10—C15—C14	2.5 (7)
C3—C2—C7—C8	159.0 (5)	C12—C11—C16—O5	160.7 (5)
C1—C2—C7—C8	−18.2 (7)	C10—C11—C16—O5	−18.7 (8)
O1—C7—C8—N1	−178.8 (4)	C12—C11—C16—N3	−19.5 (7)
C2—C7—C8—N1	2.1 (7)	C10—C11—C16—N3	161.1 (5)

### Hydrogen-bond geometry (Å, °)

**Table:**

*D*—H···*A*	*D*—H	H···*A*	*D*···*A*	*D*—H···*A*
O1—H1···O4	0.82	1.85	2.569 (5)	145
N2—H2···O5	0.86	1.92	2.607 (5)	136
N2—H2···N1	0.86	2.28	2.728 (5)	113
N3—H3A···O5^i^	0.86	2.22	2.941 (6)	141

Symmetry codes: (i) −*x*+1, −*y*+1, −*z*+1.
